# Hypermineralization of Hearing‐Related Bones by a Specific Osteoblast Subtype

**DOI:** 10.1002/jbmr.4320

**Published:** 2021-05-14

**Authors:** Yukiko Kuroda, Katsuhiro Kawaai, Naoya Hatano, Yanlin Wu, Hidekazu Takano, Atsushi Momose, Takuya Ishimoto, Takayoshi Nakano, Paul Roschger, Stéphane Blouin, Koichi Matsuo

**Affiliations:** ^1^ Laboratory of Cell and Tissue Biology Keio University School of Medicine Tokyo Japan; ^2^ Applied Cell Biology, Graduate School of Interdisciplinary Science & Engineering in Health Systems Okayama University Okayama Japan; ^3^ Institute of Multidisciplinary Research for Advanced Materials Tohoku University Sendai Japan; ^4^ Division of Materials and Manufacturing Science, Graduate School of Engineering Osaka University Osaka Japan; ^5^ Ludwig Boltzmann Institute of Osteology at the Hanusch Hospital of OEGK and AUVA Trauma Centre Meidling, 1st Medical Department Hanusch Hospital Vienna Austria

**Keywords:** BONE QCT/MICROCT, ANALYSIS/QUANTITATION OF BONE, GENETIC ANIMAL MODELS, MATRIX MINERALIZATION, BONE MATRIX, OSTEOBLASTS, CELLS OF BONE, DEVELOPMENTAL MODELING, BONE MODELING AND REMODELING

## Abstract

Auditory ossicles in the middle ear and bony labyrinth of the inner ear are highly mineralized in adult mammals. Cellular mechanisms underlying formation of dense bone during development are unknown. Here, we found that osteoblast‐like cells synthesizing highly mineralized hearing‐related bones produce both type I and type II collagens as the bone matrix, while conventional osteoblasts and chondrocytes primarily produce type I and type II collagens, respectively. Furthermore, these osteoblast‐like cells were not labeled in a “conventional osteoblast”‐specific green fluorescent protein (GFP) mouse line. Type II collagen‐producing osteoblast‐like cells were not chondrocytes as they express osteocalcin, localize along alizarin‐labeled osteoid, and form osteocyte lacunae and canaliculi, as do conventional osteoblasts. Auditory ossicles and the bony labyrinth exhibit not only higher bone matrix mineralization but also a higher degree of apatite orientation than do long bones. Therefore, we conclude that these type II collagen‐producing hypermineralizing osteoblasts (termed here auditory osteoblasts) represent a new osteoblast subtype. © 2021 The Authors. *Journal of Bone and Mineral Research* published by Wiley Periodicals LLC on behalf of American Society for Bone and Mineral Research (ASBMR).

## Graphical abstract

Type II collagen‐producing auditory osteoblasts highly mineralize hearing related bones.

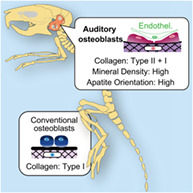

## Introduction

Auditory ossicles, including the malleus, incus, and stapes, and the bony labyrinth (also known as osseous labyrinth or otic capsule), which surrounds the cochlea, vestibule, and semicircular canals, are highly mineralized stiff bone in many mammals.^(^ ^1^ ^)^ The higher mineral density of auditory ossicles and the bony labyrinth may be relevant to hearing function because several abnormal mineralization disorders such as osteogenesis imperfecta^(^ ^2^ ^)^ and cleidocranial dysplasia^(^ ^3^ ^)^ are accompanied by hearing loss. In fact, hearing loss observed in a mouse model of cleidocranial dysplasia (*Runx2* ^+/−^ mice) and in transgenic mice with enhanced transforming growth factor‐β signaling is likely due to insufficient cochlear stiffness.^(^ ^4^ ^)^ However, how these bones are highly mineralized during development remains largely unknown.

Auditory ossicles and the bony labyrinth undergo endochondral ossification.^(^ ^5^, ^6^, ^7^, ^8^ ^)^ Unlike long bones, however, auditory ossicles and the bony labyrinth do not grow during or after ossification within the temporal bone. Using X‐ray microscopic phase imaging, we previously analyzed endochondral ossification of the mouse malleus and identified “osteogenic capillaries” composed of endothelial cells and pericytic osteoblasts.^(^ ^9^ ^)^ We also found that lumens of osteogenic capillaries gradually narrow as bone matrix is secreted onto the preexisting bone surface enclosing capillaries toward adulthood. Such close association between osteoblasts and capillaries likely supports efficient bone matrix production and mineralization during and after endochondral ossification of the malleus.

Osteoblasts produce matrix vesicles containing calcium and phosphate ions, which are biologically crystallized into apatite, the major inorganic component of bone material.^(^ ^10^ ^)^ Bone mineralization is initiated by nucleation and growth of apatite crystals in fibrillar type I collagen. The most abundant non‐collagenous protein of bone matrix, osteocalcin, tightly binds calcium ions in apatite crystal and regulates mineralization.^(^ ^11^, ^12^ ^)^ Orientation and arrangement of collagen fibrils and biological apatite has been analyzed using imaging techniques,^(^ ^13^, ^14^ ^)^ and those analyses confirm that collagen fibrils are the major determinant of bone matrix mineralization.^(^ ^12^, ^15^, ^16^, ^17^ ^)^ Thus, bone mineralization is regulated at various levels, including phosphate homeostasis and biological apatite orientation, as well as through non‐collagenous and collagenous proteins.

In this study, we assessed ossification mechanisms of highly mineralized hearing‐related bones. After analysis of auditory ossicles and the bony labyrinth in a “conventional osteoblast”‐specific *Col1a1*‐green fluorescent protein (GFP) transgenic mouse line, we identified a novel type of osteoblast that highly mineralizes bone tissue in a manner different from conventional osteoblasts. We show that both organic and inorganic components of hypermineralized ear bones exhibit properties distinct from those of conventional bones.

## Materials and Methods

### Mice

Generation of *Col1a1*‐AcGFP transgenic mice, which express AcGFP driven by the *Col1a1* promoter (2.3 kbp), was previously described.^(^ ^9^ ^)^ Mice lacking RANKL (*Tnfsf11* ^−/−^ mice)^(^ ^18^ ^)^ were on a mixed 129 and C57BL/6J background. For calcein double‐labeling, mice were subcutaneously injected with 16 mg/kg body weight calcein (FUJIFILM Wako Pure Chemical Corporation, Osaka, Japan) at P17 and P21. Mice were euthanized at P22, and bone samples were fixed in 70% ethanol. For alizarin labeling, mice were subcutaneously injected with 30 mg/kg body weight alizarin complexone (Sigma‐Aldrich, St. Louis, MO, USA) the day before euthanization. All mice were maintained under specific pathogen‐free conditions, and experiments were performed in accordance with the Institutional Guidelines on Animal Experimentation at Keio University.

### Microcomputed tomography (microCT)

MicroCT images were obtained using an X‐ray microCT scanner R_mCT2 (Rigaku Corporation, Tokyo, Japan) operated at 90 kV, 160 μA, field of view 10 mm (voxel size, 20 μm) or 30 mm (voxel size, 60 μm). Tissue mineral density (TMD) was quantified based on a phantom containing hydroxyapatite disks (200, 300, 400, 500, 600, 700, 800 mg/cm^3^) and an aluminum bar (1550 mg/cm^3^) (Ratoc System Engineering, Tokyo, Japan). X‐ray images were analyzed using Tri/3D‐BON (Ratoc System Engineering).

### Histology

The auditory bulla and capsule were isolated and fixed overnight at 4°C in 4% paraformaldehyde (PFA, TAAB Laboratories Equipment Ltd, Berkshire, UK) in PBS for paraffin blocks, or 2% PFA in PBS for frozen blocks. Detailed methods used for isolation, fixation, and embedding were previously reported.^(^ ^19^ ^)^ Only for collagen immunostaining, unfixed samples were embedded in OCT compound, and then sections were fixed with EtOH at −20°C for 5 minutes. Undecalcified frozen sections were stained with alizarin complexone (Sigma‐Aldrich). Safranin O staining was performed on decalcified paraffin sections by sequential soaking in Weigert's iron hematoxylin solution, 0.05% Fast Green solution (Polysciences, Warrington, PA, USA), 1% acetic acid solution, and 0.1% safranin O solution (Polysciences). TRAP activity was detected in paraffin sections usin*g* an Acid Phosphatase, Leukocyte Kit (387A, Sigma‐Aldrich). As primary antibodies for immunohistochemistry, we used rabbit anti‐mouse osteocalcin polyclonal antibody (1:1000, ALX‐210‐333, Enzo, Farmingdale, NY, USA), rat anti‐mouse osteocalcin antibody (5 μg/mL, R21C‐01A, Takara Bio, Shiga, Japan), rat anti‐mouse endomucin monoclonal antibody (1:50, V.7C7, Santa Cruz Biotechnology, Dallas, TX, USA), rabbit anti‐collagen type I antibody (5 μg/mL, AB765P, MilliporeSigma, Burlington, MA, USA), mouse anti‐collagen type II antibody (1 μg/mL, ab185430, Abcam, Cambridge, UK), goat anti‐SOST/Sclerostin antibody (5 μg/mL, BAF1589, R&D Systems, Minneapolis, MN, USA), and rabbit anti‐ALP antisera (1:200, a gift from Dr Amizuka). Secondary antibodies were Alexa Fluor 488/568/647‐conjugated goat anti‐rabbit/rat/mouse IgG (Thermo Fisher Scientific, Waltham, MA, USA) and Alexa Fluor 488 plus/647‐conjugated donkey anti‐ mouse/goat IgG (Thermo Fisher Scientific). For anti‐endomucin and anti‐osteocalcin antibodies, antigen retrieval was performed with 0.01 M citrate buffer (pH 6.0) at 37°C for 30 minutes in paraffin sections or with 1 μg/mL proteinase K at room temperature (RT) for 5 minutes for frozen sections. For anti‐collagen antibodies, antigen retrieval was performed with 10 μg/mL proteinase K (Takara Bio) at RT for 10 minutes in paraffin sections or without proteinase K in frozen sections. For anti‐collagen type II antibody, blocking reagent A in the Histofine MOUSESTAIN KIT (Nichirei Bioscience, Tokyo, Japan) served as blocking buffer. Nuclear counterstaining in frozen sections was performed using DAPI (diamidino‐2‐phenylindole, Sigma‐Aldrich). Sections were mounted with ProLong Diamond (Thermo Fisher Scientific) for frozen sections or Softmount (FUJIFILM Wako Pure Chemical Corporation) TrueVIEW Autofluorescence Quenching Kit (vector) for paraffin sections. Frozen and paraffin sections were observed under a confocal laser scanning microscope LSM710 (Zeiss, Thornwood, NY, USA) or a FV3000 microscope (Olympus, Tokyo, Japan) or an upright microscope BX53 (Olympus). More than three samples were stained histologically.

### Mass spectrometry

The malleus, incus, and femoral diaphysis were isolated from 3‐week‐old C57BL/6J mice (*n* = 3) and immersed in chloroform:methanol (3:1) overnight at 4°C. After washing in water, bones were decalcified in 0.1 M HCl for 3 days at 4°C. After washing in water, bones were further incubated in 4× SDS sample buffer (0.25 M Tris‐HCl, pH 6.8, 40% glycerol, 8% SDS, 20% β‐mercaptoethanol, 0.2% bromophenol blue) at 65°C for 15 minutes. Proteins in the supernatant were separated by SDS‐PAGE and stained with Coomassie Brilliant Blue. Target ~140 kDa bands were excised from each lane, followed by in‐gel digestion with 10 μg/ml trypsin (Promega, Madison, WI, USA) overnight at 37°C.^(^ ^20^ ^)^ Digested peptides were eluted in 0.1% formic acid and subjected to liquid chromatography (LC)‐MS/MS analysis on a LCMS‐IT‐TOF (Shimadzu, Kyoto, Japan) interfaced with a reverse‐phase LC system (Prominence Nano series, Shimadzu). LC was performed using a PicoFrit Beta Basic C18 column (New Objective, Littleton, MA, USA) at 300 nL/min. Peptides were eluted in a 5% to 45% acetonitrile gradient in 0.1% formic acid and sprayed into the mass spectrometer. The heated capillary was set to 200°C, and electrospray voltage was 2.5 kV. LCMS Solution software (Shimadzu) was employed for data analysis, and a text file containing observed precursor peptide *m/z*, the fragment ion *m/z*, and intensity values was obtained using Mascot Distiller (Matrix Science, Tokyo, Japan) and analyzed using Mascot MS/MS Ion Search (Matrix Science). Search parameters were: database, SwissProt; taxonomy, all; enzyme, trypsin; variable modifications, carbamidomethyl (C), oxidation (M), oxidation (P); peptide tolerance, ± 0.05 Da; and MS/MS tolerance, ±0.05 Da.

### In situ hybridization

Isolated auditory bulla and capsule (3 mice) and tibia (3 mice) were fixed with G‐Fix (GenoStaff, Tokyo, Japan) at RT for 2 days and decalcified with G‐Chelate Mild at 4°C for 2 to 3 weeks. For the latter, the buffer was changed every 3 days. Paraffin processing was performed with Cell & Tissue Processor CT‐Pro20 (GenoStaff) using ethanol and G‐NOX. Paraffin sections on CREST‐coated glass slides (Matsunami Glass, Osaka, Japan) were baked at 60°C for 1 hour, fixed with 10% formalin in PBS at 37°C for 30 minutes, treated with 0.2 M HCl at 37°C for 10 minutes, and then treated with 5 μg/mL proteinase K (FUJIFILM Wako Pure Chemical Corporation) in PBS for 10 minutes at 37°C. Digoxigenin (DIG)‐labeled RNA probes (mouse *Col1a1*, *Col2a1*, *Runx2*, *Sp7*; GenoStaff) were diluted to 250 ng/mL with G‐Hybo‐L buffer and hybridized at 60°C for 20 hours under coverslips. After removing coverslips in 1× G‐Wash, sections were washed once with 1× G‐Wash at 60°C for 10 minutes, once with 50% formamide in 1× G‐Wash at 60°C for 10 minutes, twice with 1× G‐Wash at 60°C for 10 minutes, twice with 0.1× G‐Wash at 60°C for 10 minutes, and twice with Tris buffered saline (50 mM Tris pH 7.5, 150 mM NaCl) containing 0.1% Tween 20 (TBST). After G‐Block treatment for 15 minutes, sections were incubated with Fab fragments from anti‐DIG antibody conjugated to alkaline phosphatase (Roche, Mannheim, Germany) diluted 1:2000 in 0.02× G‐Block/TBST for 1 hour. Sections were washed twice with TBST and then incubated in AP buffer (100 mM NaCl and 100 mM Tris–HCl, pH 9.5). The color reaction was performed with NBT/BCIP stock solution (Roche) diluted 1:50 in AP buffer and stopped with PBS. Sections were counterstained with Mayer's hematoxylin solution (FUJIFILM Wako Pure Chemical Corporation), mounted with G‐mount, and observed microscopically (BX53, Olympus).

### Synchrotron radiation X‐ray phase imaging

Mallei (*n* = 4) were isolated as described^(^ ^19^ ^)^ and fixed with 2% glutaraldehyde (FUJIFILM Wako Pure Chemical Corporation), 2% PFA in HEPES buffer (30 mM HEPES, 100 mM NaCl, 4 mM CaCl_2_, pH 7.4) at RT for 2 hours. After washing with HEPES buffer, mallei were dehydrated in an ethanol series and dried in a critical point dryer (Leica EM CPD300). Talbot phase‐sensitive X‐ray tomographic microscope images were obtained using a monochromatic X‐ray beam (9.0 keV) at the beamline BL37XU of SPring‐8 (for Super Photon ring‐8 GeV) (Hyogo, Japan), as described.^(^ ^21^, ^22^, ^23^ ^)^ Conditions were 10 seconds/projection and 720 projections/180 degrees, and X‐ray imaging optical magnification was 102 (voxel size, 0.2 μm). X‐ray images were analyzed using Tri/3D‐BON (Ratoc System Engineering) and ImageJ software (NIH). Types of cells were identified by morphological comparison of X‐ray images and corresponding histological sections.

### Bone mineral content, mineral apposition rate, and analysis of the osteocyte network

Before embedding in polymethylmethacrylate, femur and mallei were bulk‐stained with rhodamine as described.^(^ ^24^ ^)^ Subsequently, bone sample blocks were trimmed using a diamond saw. The surface containing the sectioned bone area was ground and polished using a water‐free procedure. After carbon coating, samples were analyzed using a field emission scanning electron microscope (FESEM) (Zeiss Supra 40, Oberkochen, Germany) equipped with a four‐quadrant semiconductor backscattered electron detector. The FESEM was operated with an electron energy of 20 keV. Quantitative backscattered electron imaging (qBEI) was performed on femoral cross sections (*n* = 4) and malleus sections (*n* = 8) through the mPb. Calibration was performed using the “atomic number contrast” of reference materials. The degree of mineralization in terms of the calcium weight fraction (weight‐percent Ca, weight% Ca) was quantified with a 0.88 μm/pixel spatial resolution using the atomic number contrast of carbon (Z = 6) and aluminum (Z = 13) as reference materials.^(^ ^25^ ^)^ After qBEI, the same surfaces were investigated using confocal laser scanning microscopy CLSM (Leica TCS SP5) equipped with an oil immersion lens (Leica, HCX PL APO 63x NA 1.25 OIL). The 488 nm line (Argon laser) was chosen for observation of calcein double‐labeling at the sample surface. Subsequently, we obtained 3D image stacks of the osteocyte lacuno‐canalicular network using the 543 nm line (HeNe laser) for rhodamine excitation. The fluorescence signal was measured in a spectral window between 553 and 705 nm with the airy 1 pinhole of 67.93 μm (spatial resolution of 0.24 μm/pixel).

### Transmission electron microscopy (TEM)

Mallei and femurs were isolated from 3‐week‐old C57BL/6J mice and fixed with 2% glutaraldehyde (FUJIFILM Wako Pure Chemical Corporation) and 2% PFA in HEPES buffer (30 mM HEPES, 100 mM NaCl, 4 mM CaCl_2_, pH 7.4) overnight at 4°C. After washing with HEPES buffer, samples were decalcified with 10% EDTA/0.1 M Tris, pH 7.0, at 4°C for 3 weeks and treated with 1% tannic acid (FUJIFILM Wako Pure Chemical Corporation) in HEPES buffer at RT for 2 hours. After 2 hours of post‐fixation with 1.0% OsO_4_ (TAAB Laboratories Equipment, Aldermaston, UK), samples were treated with an EtOH series (70% to 100%), acetone (Sigma‐Aldrich), n‐butyl glycidyl ether (QY‐1; Okenshoji, Tokyo, Japan), and then graded concentrations of Epon (Okenshoji) by QY‐1. Finally, samples were incubated in 100% Epon 72 hours at 4°C to enhance resin penetration. After 72 hours of polymerization in 100% Epon at 60°C, ultrathin sections (70 nm thickness) were prepared using an ultramicrotome (Leica UC7; Leica Biosystems, Wetzlar, Germany) with a diamond knife and collected every 5 slices on copper grids. Sections were imaged by TEM (JEM‐1400plus, JEOL, Tokyo, Japan). To determine collagen fibril diameter, TEM images (*n* = 45 of femur and *n* = 9 of mallei) were analyzed using Fiji (distributed by ImageJ, an open‐source Java image processing program developed by NIH). The FFT Bandpass Filter, which sets a structure size at 10 to 300 pixels (TEM image resolution; 1.47 pixels/nm), was applied and brightness was automatically adjusted. Diameters were measured as a minor axis of ellipses using Analyze Particles. Values were set to 600‐infinity pixel^2^ (size) and 0.80 to 1.00 (circularity).

### Analysis of preferential orientation of the apatite *c*‐axis

The degree of preferential apatite *c‐*axis orientation was analyzed by employing a microbeam X‐ray diffractometer (μXRD) (R‐Axis BQ; Rigaku Corporation) with a transmission optical system and Mo target. A Mo‐Kα line was generated at 50 kV and 90 mA. Double‐pinhole metal collimators with diameters of 200 and 800 μm were used, and μXRD measurements were carried out at points indicated by arrows shown in Fig. 7*A*, *B*. For the femur (4 mice), the incident beam was radiated vertically to the specimen at the center of the bone width. For the malleus (4 mice), to avoid X‐ray irradiation of the lamina, the beam center was displaced anteriorly, excluding the lamina.^(^ ^26^ ^)^ The diffracted beam was counted for 10 ks for malleus and 300 s for femur by an imaging plate (Fuji Film, Tokyo, Japan). The preferential orientation degree of the biological apatite *c‐*axis was evaluated by calculating the intensity ratio of (002)/(310), which is a dimensionless index.^(^ ^14^, ^27^ ^)^ This value for randomly oriented apatite (National Institute of Standards and Technology, Standard Reference Material 2910: calcium hydroxyapatite) was confirmed to be 0.6. Thus, detected values >0.6 indicate an anisotropic apatite *c‐*axis orientation in the direction analyzed.

### Statistical analysis

All experiments were performed at least three times. Where appropriate, the number of mice or biological replicates is given in figure legends. Statistical analyses were performed using Mann–Whitney *U* test or Student's *t*‐test on SPSS software version 26 (IBM Corp., Armonk, NY, USA). A *p* < 0.05 was considered statistically significant.

## Results

### Auditory ossicles and the bony labyrinth are highly mineralized in adult and young mice

**Fig 1:**
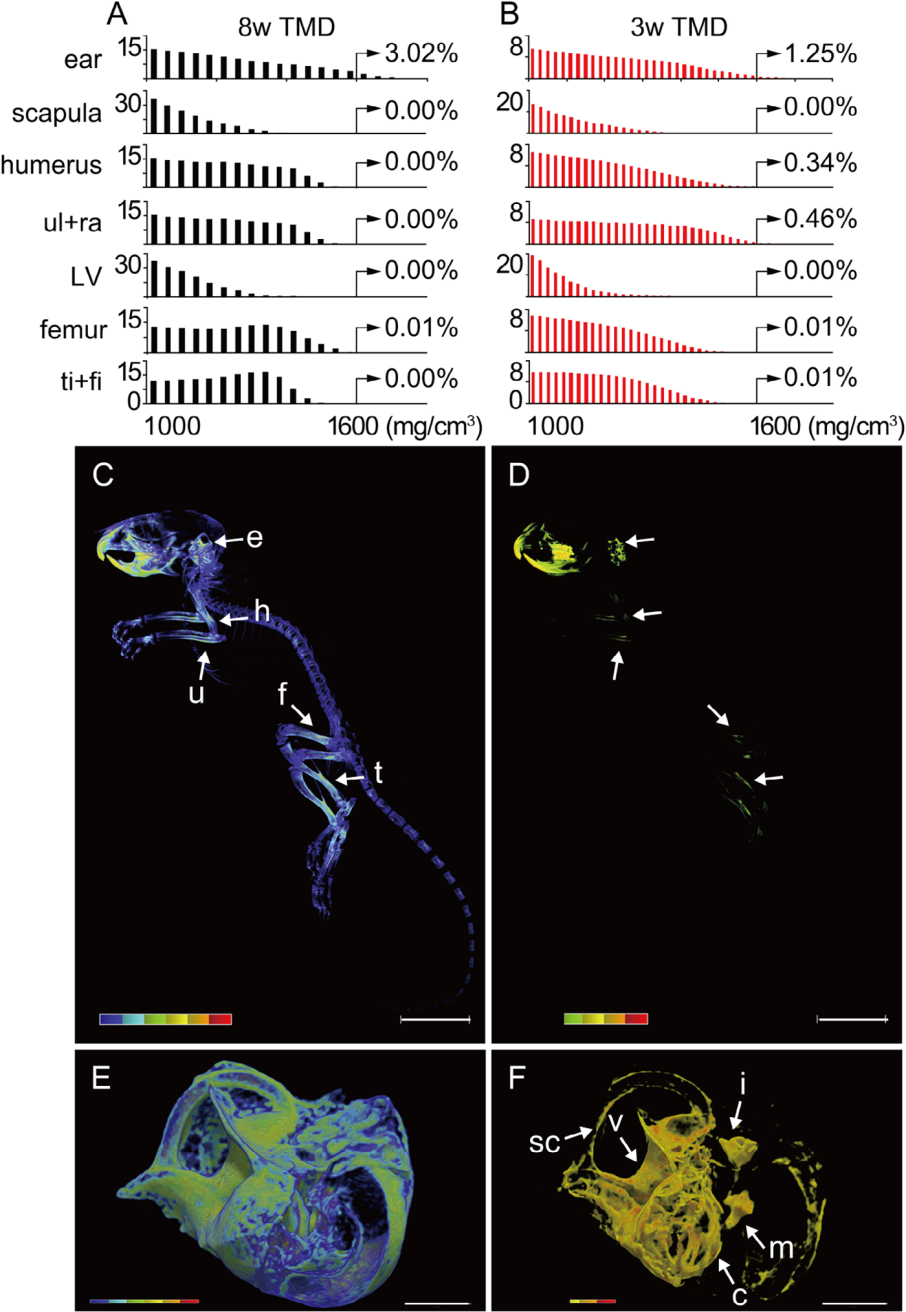
Hearing‐related bones are highly mineralized in adult and young mice. (*A*) Frequency histograms of tissue mineral density (TMD) of each bone at 8 weeks of age. Shown is percentage of TMD voxels above 1000 mg/cm^3^. Voxel size = 20 μm. ul + ra = ulna and radius; LV = lumbar vertebra; ti + fi = tibia and fibula. (*B*) Frequency histograms of TMD of each bone isolated from P21 mouse shown in (*C*). Voxel size = 10 μm. (*C*) Heat map of TMD (100–1500 mg/cm^3^) in a microCT image of the whole body of a P21 mouse. Voxel size = 60 μm. Arrows indicate ear bones (e), humerus (h), ulna (u), femur (f), and tibia (t). Scale bar = 10 mm. (*D*) High TMD regions (500–1500 mg/cm^3^) of (*C*). (*E*) TMD heat map (100–1800 mg/cm^3^) of left ear bones (anterior view; lateral is to the right). Voxel size = 10 μm. Scale bar = 1 mm. (*F*) High TMD regions (1000–1800 mg/cm^3^) of (*E*). Arrows indicate malleus (m), incus (i), cochlea (c), vestibule (v), and semicircular canals (sc).

We first performed microCT imaging to confirm whether hearing‐related bones are highly mineralized stiff bone in adult mouse like many mammals. Tissue mineral density (TMD) histograms of each whole bone in adult (8‐week‐old) mice revealed a characteristic TMD distribution with the ear bone exhibiting the greatest TMD regions (Fig. 1*A* and Supplemental Fig. S1*A*, *B*). Since the bones of ear include low and high TMD regions, we focused on the high TMD bones in the ear, such as auditory ossicles and bony labyrinth (Supplemental Fig. S1*C*, *D*). The average TMD of malleus is significantly higher than that of femoral mid‐diaphysis (Supplemental Fig. S1*E*), indicating that hearing‐related bones are highly mineralized in adult mice. To assess timing of hypermineralization of ear bones during development, we measured TMD on mice at postnatal day 21 (P21), a time when auditory ossicles undergo rapid bone formation^(^ ^9^ ^)^ (Fig. 1*B–D*). Bones of the ear, humerus, ulna, femur, and tibia exhibited TMD higher than 500 mg/cm^3^ (Fig. 1*D*) and bones of the ear showed the greatest TMD (Fig. 1*B*). Parts of the middle and inner ear bones showing TMD >1000 mg/cm^3^ included auditory ossicles (middle ear), such as the malleus and incus, and the bony labyrinth (inner ear) containing the cochlea, vestibule, and semicircular canals (Fig. 1*E*, *F*). These data indicate that hearing‐related bones in weaning mice already exhibit hypermineralization and that the ossification process of these bones may differ from that of other bones.

### Ossification of auditory ossicles by osteogenic capillaries

**Fig 2:**
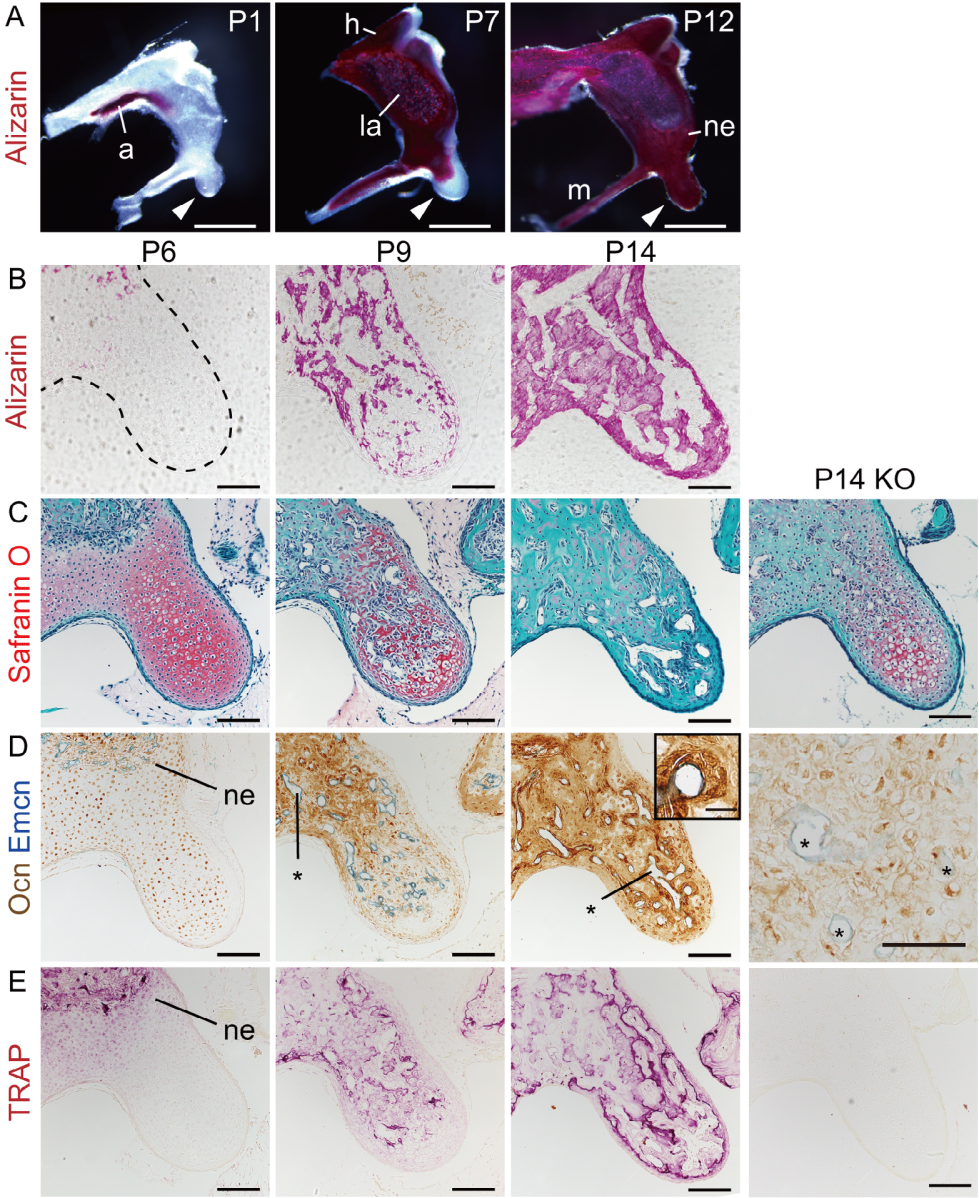
Ossification of auditory ossicles by osteogenic capillaries. (*A*) Whole‐mount alizarin red staining of mallei at indicated stages. Scale bars = 500 μm. a = anterior process; h = head; la = lamina; ne = neck; m = manubrium. Arrowheads = mPb. (*B*) Alizarin complexone staining of mineralizing bone matrix (red) in undecalcified frozen sections of the mPb dashed line outlines the cartilaginous mPb. Scale bars = 100 μm. (*C*–*E*) Paraffin sections of the mPb from wild‐type and P14 RANKL KO (P14KO) mice. (*C*) Safranin O staining of cartilage (red) and bone (green). Scale bars = 100 μm. (*D*) Osteocalcin (Ocn; brown)‐positive osteoblasts and endomucin (Emcn; blue)‐positive endothelial cells. Asterisks = capillary lumen. Scale bars = 100 μm in P6, P9, and P14; 20 μm in magnified view of P14; and 50 μm in P14 KO. (*E*) TRAP‐activity staining (red) of chondro/osteoclasts. At P14, TRAP‐positive cement lines are visible under the thin cortex in wild‐type mice. Scale bars = 100 μm.

We next examined developmental ossification processes of the malleus. To determine the time course of mineralization, we undertook alizarin staining of bone matrix in the malleus from P1 to P12. This analysis, shown in Fig. 2*A*, revealed that the cartilaginous malleus undergoes endochondral ossification starting at the anterior process and proceeding to the head, lamina, neck, manubrium, and finally toward the tip into the malleal processus brevis (mPb; arrowheads), observations consistent with a previous report.^(^ ^28^ ^)^ Histological analysis of the mPb showed that the process of replacing cartilage with alizarin‐positive bone matrix was almost complete by P14 (Fig. 2*B*). Reciprocally, safranin O‐positive cartilage matrix observed at P6 was fully removed by P14 (Fig. 2*C*). Endomucin (Emcn)‐positive capillary vessels were detectable along with surrounding osteocalcin (Ocn)‐positive mature osteoblasts in alizarin‐positive areas (Fig. 2*D*). Specifically, narrow endomucin‐positive capillaries entered around the neck of malleus at P6 had reached the mPb at P9, when the capillary lumen in the neck had become broader. By P14, endomucin‐positive capillary vessels with osteocalcin‐positive osteoblasts were detectable in the entire mPb, and the lumens of these capillaries showed a greater diameter than that found at P9. The diameter of capillary vessels in the neck of malleus at P14 decreased relative to that found at P9 (Fig. 2*D*) illustrating the sequence of events: The cartilaginous mPb is first invaded by narrow capillary vessels, whose lumens then undergo enlargement. Enlarged capillary vessels form “osteogenic capillaries” composed of endothelial cells and osteocalcin‐positive pericytic osteoblasts, which deposit alizarin‐positive mineralized bone matrix around osteogenic capillaries, thereby narrowing capillary lumens as ossification proceeds inward. This narrowing of capillaries continues to adulthood.^(^ ^9^ ^)^

Chondroclasts, which are specialized macrophages in the same lineage as osteoclasts, remove cartilage matrix to allow new bone matrix to be laid down by osteoblasts. When we examined the P6 mPb, we detected tartrate‐resistant acid phosphatase (TRAP)‐positive chondroclasts at the ossification front in the neck invaded by narrow blood vessels and observed chondro/osteoclasts around enlarged capillary lumens at P9 and P14 (Fig. 2*E*). We next asked whether chondroclasts were required for formation of osteogenic capillaries in the ossifying malleus. To this end, we analyzed the malleus of *Tnfsf11* ^−/−^ (RANKL KO) mice, which lack chondro/osteoclasts.^(^ ^18^ ^)^ At P14, when most cartilage matrix is resorbed in wild‐type mice (Fig. 2*C*), safranin O‐positive cartilage matrix remained unresorbed in RANKL KO mice, and capillary lumens were not enlarged in the mPb (Fig. 2*C*; P14 KO). At that same time point, osteocalcin‐positive mature osteoblasts had not appeared around capillary vessels in KO mice (Fig. 2*D*; P14 KO), although alkaline phosphatase‐positive preosteoblasts had accumulated around endomucin‐positive capillary vessels (Supplemental Fig. S2). These data suggest that osteogenic capillaries in auditory ossicles require the presence of chondroclasts in order for preosteoblasts to terminally differentiate into osteocalcin‐positive osteoblasts at the basement membrane of capillary vessels.

### Analysis of 3D morphological changes in auditory ossicles during endochondral ossification

**Fig 3:**
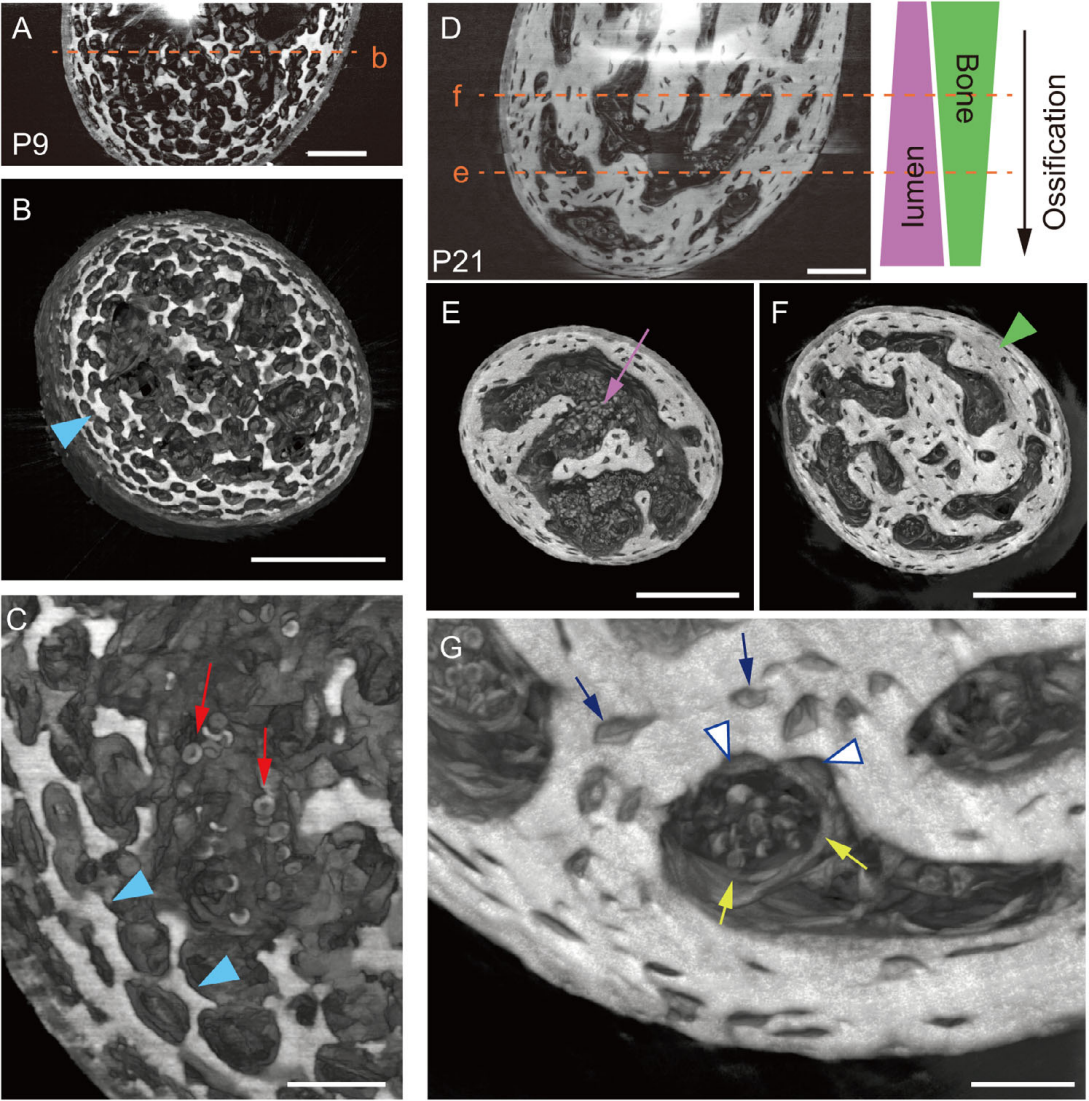
Synchrotron X‐ray phase images of mPb during endochondral ossification. (*A*) Mineralized lattice‐like cartilaginous matrix in the P9 mPb. Vertical section. Dotted line b indicates the horizontal cutting plane shown in (*B*). Scale bar = 50 μm. (*B*) Horizontal section showing partially resorbed cartilaginous matrix (arrowhead). Scale bar = 100 μm. (*C*) Magnified view of (*A*) showing capillary invasion. Arrows = red blood cells; arrowheads = cartilaginous matrix. Scale bars = 20 μm. (*D*) Mineralized bone matrix in the P21 mPb. Vertical section. Dotted lines e and f indicate horizontal cutting planes shown in (*E*) and (*F*), respectively. Scale bar = 50 μm. (*E*) Horizontal section near the tip of mPb, showing enlarged osteogenic capillary lumen (arrow). Scale bar = 100 μm. (*F*) Horizontal section of the middle of mPb, showing osteogenic capillaries with narrower lumens surrounded by more increased bone matrix (arrowhead) than in (*E*). Scale bar = 100 μm. (*G*) Magnified view of osteogenic capillaries in (*F*). Yellow arrows = endothelial cells; open arrowheads = osteoblasts; blue arrows = osteocytes. Cell types were identified by morphology and proximity to red blood cells. Scale bar = 20 μm.

To depict cartilage, bone, and soft tissues in the same view, we performed Talbot phase‐contrast X‐ray synchrotron tomography, which allows quantitative imaging of material density.^(^ ^21^, ^23^ ^)^ At P9, mineralized lattice‐like cartilaginous matrix was partially resorbed in mPb as expected (Fig. 3*A*, *B*). Higher magnification revealed capillary vessels, which contained red blood cells, had invaded cartilaginous matrix (Fig. 3*C*). By P21, the entire mPb had been replaced by bone matrix (Fig. 3*D*) and osteogenic capillaries were narrowing as bone rapidly formed (Fig. 3*E*, *F*). We also observed that osteoblasts adjacent to endothelial cells were in contact with bone matrix and that osteocytes were embedded in bone matrix around osteogenic capillaries (Fig. 3*G* and Supplemental Fig. S3). Morphological analyses so far suggest that, except for high TMD and osteogenic capillary formation, osteoblasts of auditory bones look like genuine osteoblasts because they deposit bone matrix and become osteocytes.

### Osteoblasts in auditory ossicles and the bony labyrinth express *Col2a1*

**Fig 4:**
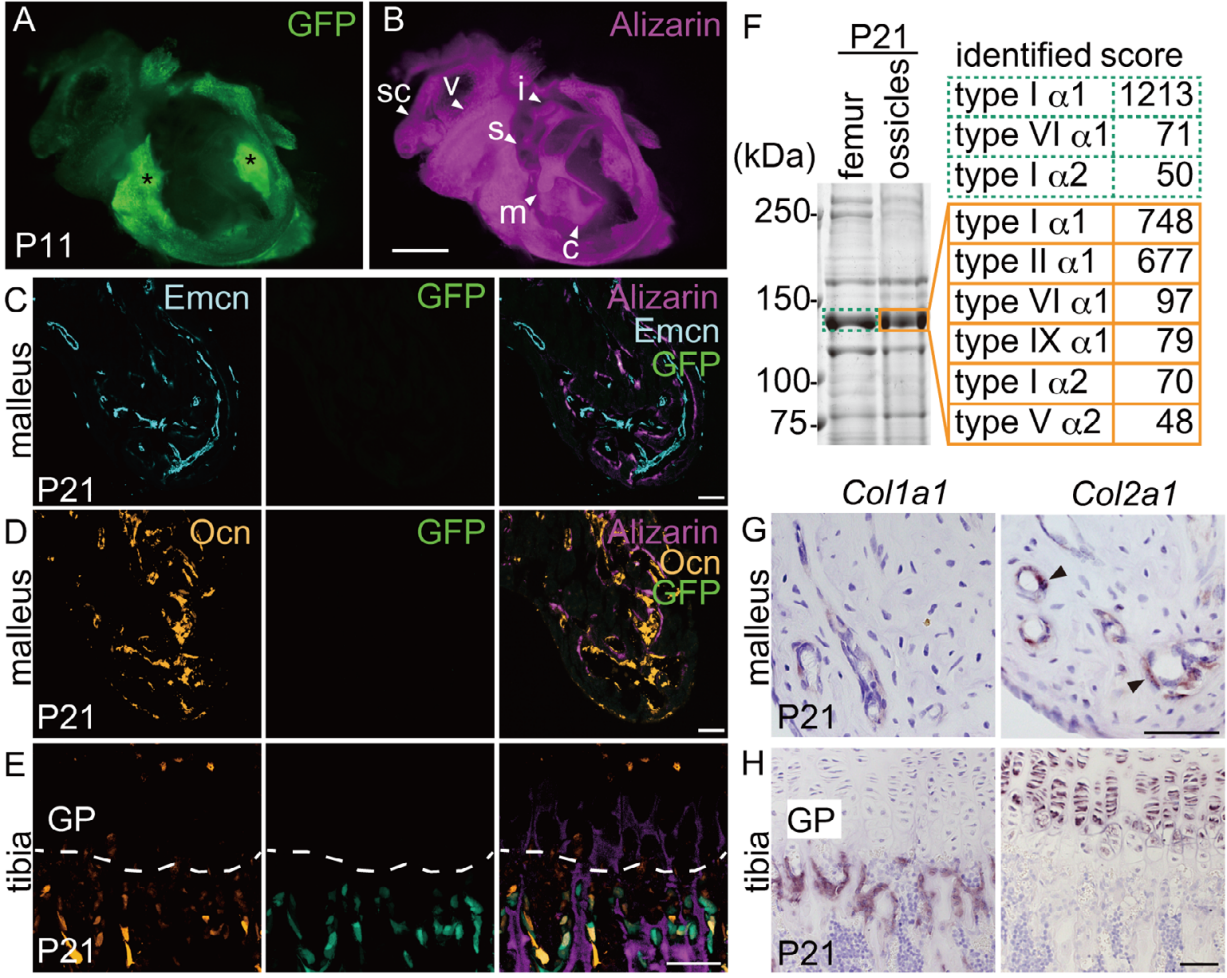
Osteoblasts in auditory ossicles and the bony labyrinth express *Col2a1*. (*A*) GFP signals of ear bone containing ossicles and the bony labyrinth of a GFP^OB^ mouse at P11. Ossicles and bony labyrinth are GFP‐negative, while tympanic bulla is GFP‐positive (asterisks). (*B*) Alizarin‐labeled bone matrix of (*A*). Scale bar = 1 mm. s = stapes. See Fig. 1 for other abbreviations. (*C*) Emcn‐positive endothelial cells, GFP signals, and a merge of alizarin, Emcn, and GFP signals in the malleus of a P21 GFP^OB^ mouse. Scale bar = 50 μm. (*D*, *E*) Ocn‐positive and GFP‐positive cells, and a merge of alizarin, Ocn, and GFP signals in the malleus (*D*) or the proximal tibial chondro‐osseous junction (*E*, dashed line) of a P21 GFP^OB^ mouse. GP = growth plate. Scale bar = 50 μm. (*F*) Mass spectrometry analysis of collagen components of femur and auditory ossicles. Boxed 140 kDa bands were excised and analyzed. (*G*, *H*) In situ hybridization for *Col1a1* and *Col2a1* in the malleus (*G*) or tibia (*H*) of P21 mouse. Arrowheads = *Col2a1*‐positive cells. Scale bars = 50 μm.

To further characterize osteoblasts that deposit bone matrix around osteogenic capillaries in hypermineralized bones of the ear, we analyzed fluorescent signals in GFP transgenic mice (the *Col1*‐GFP mouse, hereafter called the GFP^OB^ mouse), in which conventional osteoblasts are marked by GFP.^(^ ^29^, ^30^ ^)^ Unexpectedly, GFP signals were absent in auditory ossicles (malleus, incus, and stapes) and the bony labyrinth (cochlea, vestibule, and semicircular canals) at P11 (Fig. 4*A*), although alizarin labeling confirmed that active osteogenesis was occurring in these bones at this stage (Fig. 4*B*). At P21, analysis of the mPb of GFP^OB^ mice showed alizarin labeling indicative of bone formation along endomucin‐positive osteogenic capillaries in the absence of GFP‐positive osteoblasts (Fig. 4*C*). Also at P21, we observed abundant osteocalcin‐positive osteoblasts in the mPb but detected no GFP‐positive osteoblasts in this area (Fig. 4*D*). By contrast, in the tibia of the same mouse, we detected GFP and osteocalcin double‐positive osteoblasts at alizarin‐labeled bone formation sites below growth plate (Fig. 4*E*). These results suggest that the GFP‐negative osteoblast‐like cells in auditory ossicles and the bony labyrinth represent a new type of osteoblasts, which we termed here “auditory osteoblasts.”

Because GFP^OB^ mice carry a *Col1* promoter‐driven GFP transgene, we asked what are the collagen subtypes produced by auditory osteoblasts. We extracted proteins from demineralized femoral diaphyses and auditory ossicles of P21 mice. Overall patterns of protein bands found in Coomassie‐stained SDS‐PAGE gels were similar in ossicles and femur (Fig. 4*F*). Given that the α1 chains of collagens type I, type II, and type III among fibrillar collagens are approximately 140 kDa, we asked whether the prominent 140 kDa band found in both ossicle and femur samples contained different components. Mass spectrometry of the 140 kDa band from femoral diaphysis identified type I collagen, as expected; however, the comparable band from auditory ossicles contained both type I and type II collagens (Fig. 4*F*). This latter finding was unexpected, as in mouse ossicles, cartilage matrix containing type II collagen is almost fully replaced with bone matrix by as early as P14 except for the joint (Fig. 2*A*). To determine whether auditory osteoblasts express *Col2a1* (encoding the α1 chain of type II collagen), we next used in situ hybridization. In the malleus at P21, *Col1a*‐positive osteoblasts, which were also positive for *Runx2* and *Sp7*, around capillary lumens clearly expressed *Col2a1* (Fig. 4*G* and Supplemental Fig. S4). By contrast, at the tibial metaphysis, *Col1a1*‐positive osteoblasts did not express *Col2a1*, while, as expected, proliferating chondrocytes expressed *Col2a1* (Fig. 4*H*). These data indicate that auditory osteoblasts, but not conventional osteoblasts, express *Col2a1*.

### Auditory ossicles and the bony labyrinth contain type II collagen as bone matrix

**Fig 5:**
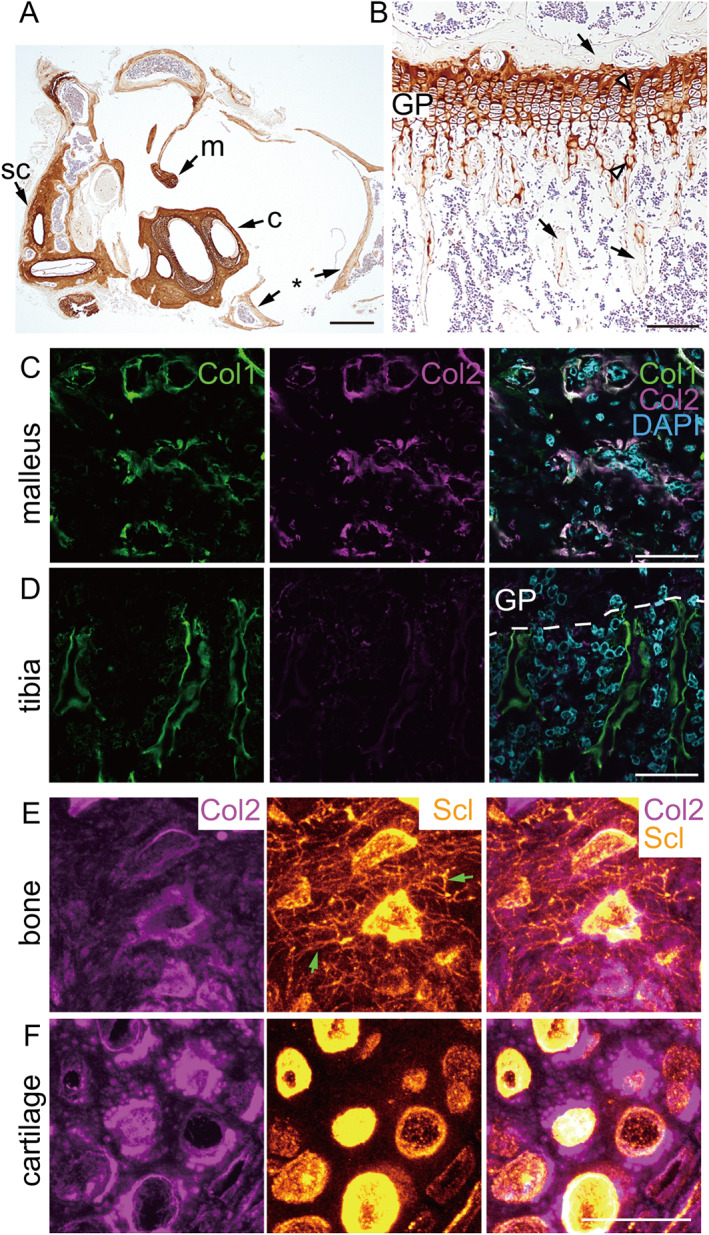
Hearing‐related bones contain type II collagen as bone matrix. (*A*) Immunolocalization of type II collagen in paraffin sections of middle and inner ear of a 12‐week‐old mouse. Arrows = malleus (m), cochlea (c), semicircular canals (sc), and tympanic bulla (asterisk). Scale bar = 500 μm. (*B*) Immunolocalization of type II collagen in paraffin sections of the proximal chondro‐osseous junction of the tibia of an 8‐week‐old mouse. Arrows = bone; arrowheads = cartilage. Scale bar = 500 μm. (*C*, *D*) Double immunostaining of type I (Col1) and type II (Col2) collagen in undecalcified frozen sections of the malleus (*C*) and femur (*D*) at P14. Scale bars = 50 μm. (*E*, *F*) Type II collagen (Col2) and Sclerostin (Scl) double‐positive cells in the bone matrix (*E*) or articular cartilage (*F*) of P21 malleus. Note that osteocytes, but not chondrocytes, extend canaliculi network (arrows).

We next performed immunostaining with anti‐type II collagen antibody to confirm whether highly mineralized adult ear bone consists of type II collagen. The malleus and bony labyrinth showed robust type II collagen staining (Fig. 5*A*), as did control tibial growth plate cartilage (Fig. 5*B*). By contrast, ordinary bone matrix in the tympanic bulla (Fig. 5*A*, asterisk) and tibia (Fig. 5*B*, arrows) was type II collagen‐negative. Double‐immunostaining for both type I and type II collagens also revealed that type I collagen‐positive osteoblasts in the malleus contained type II collagen (Fig. 5*C*), whereas those in femoral bone did not (Fig. 5*D*). Both osteocytes and articular chondrocytes were sclerostin‐positive in the malleus as is the case in long bones.^(^ ^31^ ^)^ Osteocytes in the malleus, which expressed type II collagen, were interconnected through sclerostin‐positive canaliculi network as conventional osteoblasts, while articular chondrocytes, which also expressed type II collagen, were devoid of such network (Fig. 5*E*, *F*). These results revealed that auditory osteoblasts and osteocytes express type II collagen and that hearing‐related bones are type II collagen‐rich bones.

### High‐calcium bone contains small‐diameter fibrils

**Fig 6:**
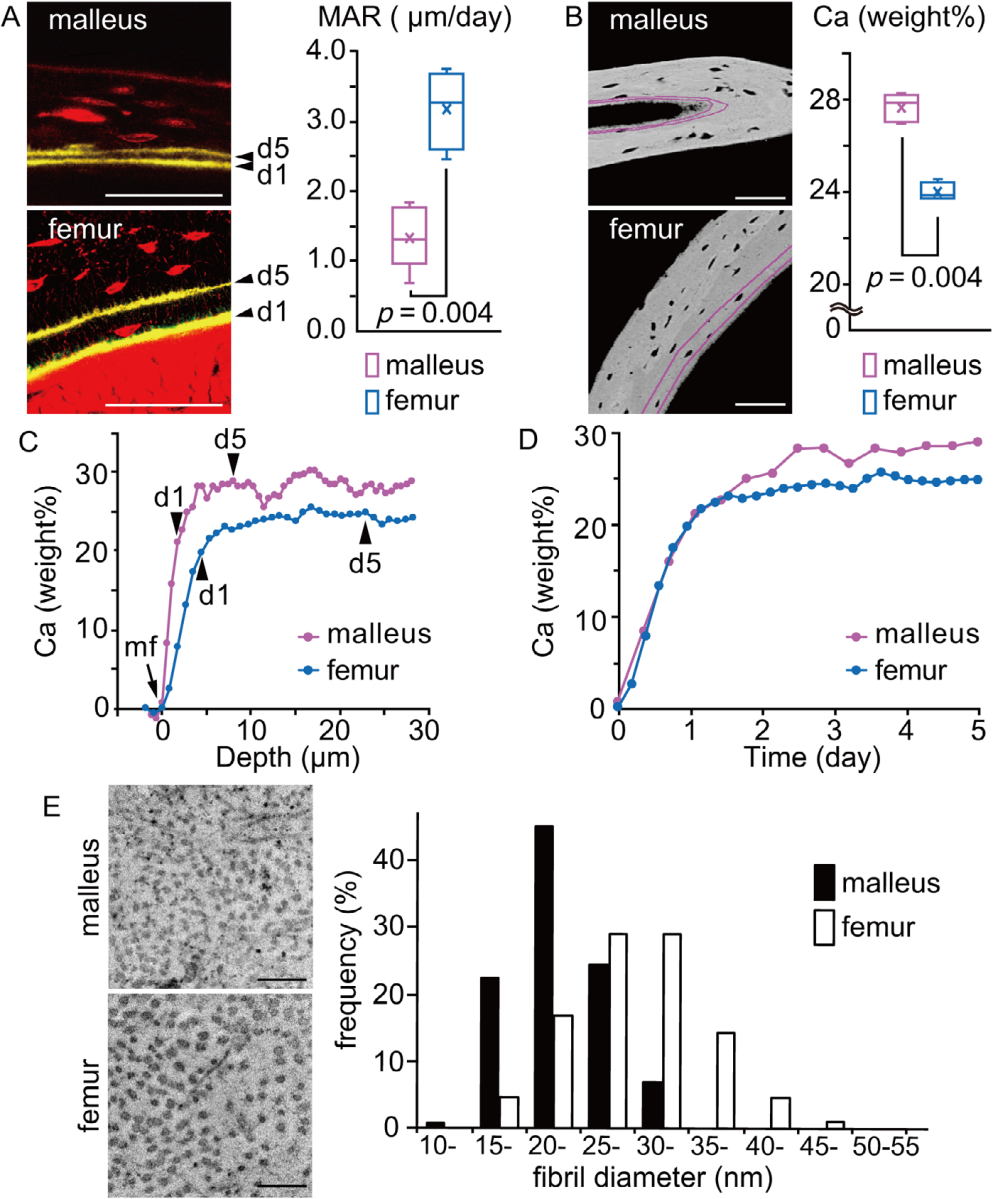
Highly mineralized bone contains small diameter fibrils. (*A*) Mineral apposition rate (MAR) in malleus (*n* = 8) and femur (*n* = 4) of P21 mice. Yellow lines indicate calcein double‐labeling at days 1 and 5. Scale bars = 50 μm. Mann–Whitney *U* test, *p* = .004; x = mean value. (*B*) Calcium concentration (Ca weight%) in malleus (*n* = 8) and femur (*n* = 4) of P21 mice, quantified using qBEI images in the area between calcein double‐labeling (magenta lines). Scale bars = 50 μm. Mann–Whitney *U* test, *p* = .004; x = mean value. (*C*) Bone mineralization profiles of the malleus and femur of P21 mice. The *x*‐axis shows the depth of mineralized bone matrix from the mineralization front (mf). Arrowheads indicate positions of calcein double‐labeling at days 1 and 5. (*D*) Mineralization time course based on MAR and bone mineralization profiles. The *x*‐axis shows estimated time in days. (*E*) Left: Ultrastructure of collagen fibrils in cross sections of malleus and femur of P21 mice. Scale bars = 200 nm. Right: Collagen fibril diameter distribution in malleus (*n* = 102) and femur (*n* = 586). Note that distribution in the malleus covered a significantly smaller range than did that of femur. Mann–Whitney *U* test, *p* < .001.

To assess properties of bone made by auditory osteoblasts, we investigated bone mineral content by quantitative backscattered electron imaging (qBEI) combined with confocal scanning laser microscopy. To mark the mineralization front, calcein was injected twice into mice at 5 days and 1 day before euthanization at P21. The mineral apposition rate (MAR), defined as the average distance between fluorescent double‐labels divided by the time interval between two calcein injections, was significantly lower in the malleus relative to the femur in P21 mice (Fig. 6*A*), indicating that less bone material was deposited in the malleus compared with the femur over the same time interval. Calcium concentration (weight%) in newly formed bone (between labels) was much higher in malleus than in femur in the same mouse at the same time after new bone formation (Fig. 6*B*). Furthermore, to analyze the bone mineralization profile of newly formed bone, we plotted Ca (weight%) against the depth of mineralized bone matrix from the mineralization front (Fig. 6*C*). That analysis showed that less osteoid is produced overall in the malleus than in femur, but the malleus exhibits higher levels of Ca (weight%) than femur at the same distance from the mineralizing surface. This observation was confirmed when we converted the *x*‐axis into time (days) by dividing the depth (μm) by MAR (μm/d) (Fig. 6*D*). That analysis indicated a comparable rate of calcium deposition between malleus and femur at an initial rapid phase (marked by a steep slope) of mineralization (primary mineralization); however, over a prolonged period of secondary mineralization marked by flattening of the curve, mineral content (Ca weight%) was higher in malleus than in femur. These results suggest that bone matrix produced by auditory osteoblasts is of higher mineral content than ordinary bone matrix.

We next observed ultrastructure of collagen fibrils in osteoid adjacent to osteoblasts and compared their diameter in malleus and femoral cortical bone (Fig. 6*E*). In the malleus, that diameter was significantly smaller than that found in the femur, an observation consistent with the fact that type II collagen fibrils are thinner than those of type I.^(^ ^32^ ^)^ Since the diameter of type I collagen fibrils increases with higher degrees of mineralization,^(^ ^33^ ^)^ we conclude that thin fibrils found in hypermineralized malleus represent type II collagen.

### Bone formed by auditory osteoblasts shows a high degree of apatite orientation

**Fig 7:**
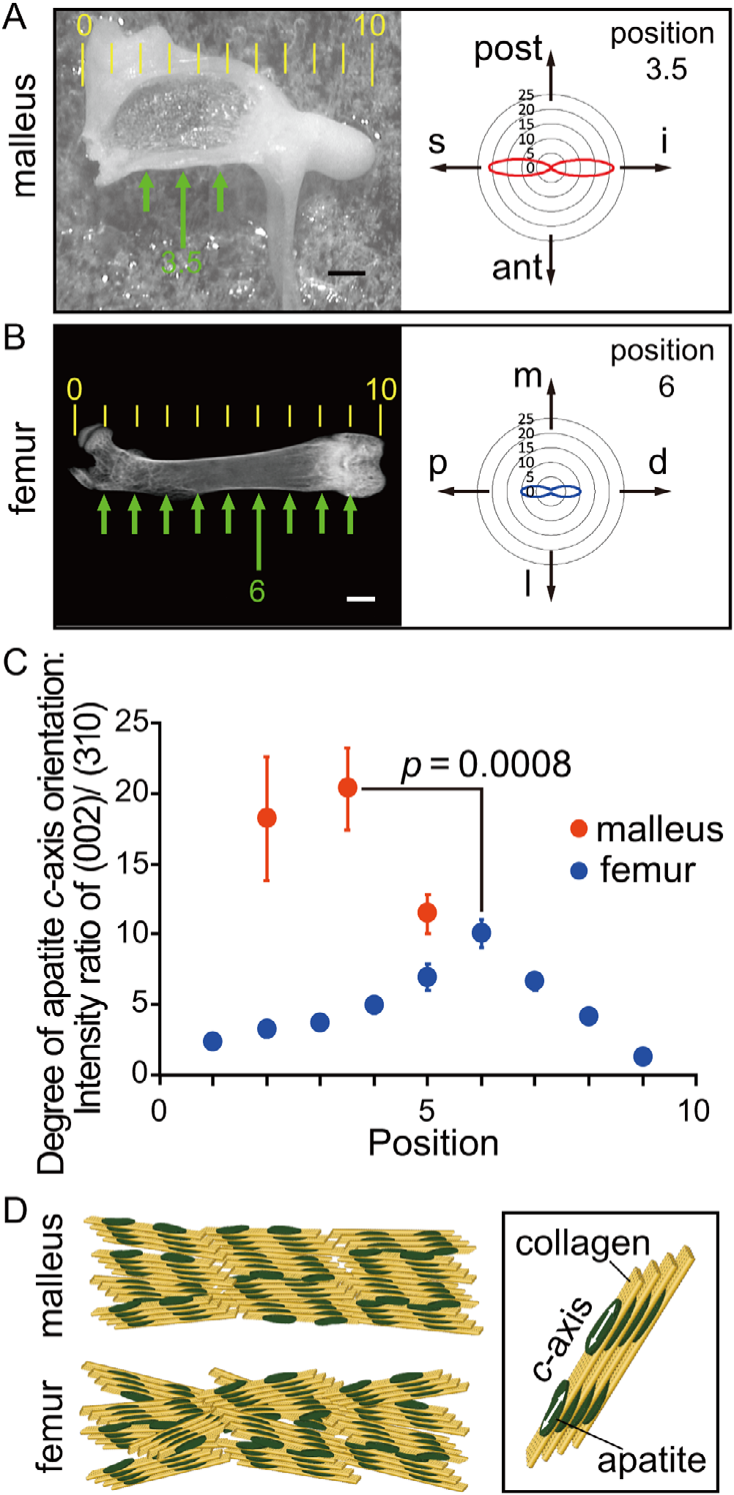
The malleus shows a high degree of apatite orientation. (*A*, *B*) Left: Representative images of malleus (*A*) and femur (*B*) showing points (green arrows) assessed for μXRD. Scale bars = 200 μm (malleus) and 1 mm (femur). Full bone lengths in (*A*) and (*B*) were divided into 10 equal parts (indicated by yellow ruler). Right: Polar diagrams representing the degree of apatite *c*‐axis orientation at peak positions (indicated by long green arrows). The radius represents the intensity ratio of (002) and (310) diffraction peaks over 360°: the greater the diameter, the more prominent the orientation. ant = anterior; post = posterior. s = superior; i = inferior; m = medial; l = lateral; p = proximal; d = distal. (*C*) Plot of the maximum degree of *c*‐axis orientation in malleus (*n* = 4) or femur (*n* = 4) at positions indicated by green arrows in (*A*, *B*). Error bars represent SD. Student's *t*‐test, position 3.5 of malleus versus position 6 of femur, *p* < .01. (*D*) Schematic diagram of the alignment of apatite *c*‐axis and collagen fibrils in malleus and femur. In the malleus, the *c*‐axis of apatite crystals on collagen fibrils is more highly aligned to the bone axis than it is in femur. The *c*‐axis orientation on collagen fibrils is shown in the box at the right.

To compare the nanostructure of malleus relative to femoral cortical bone, we examined the preferential orientation of biological apatite crystallites using a microbeam X‐ray diffraction system. Because biological apatite crystallizes on the collagen template and grows via self‐assembly almost parallel to the longitudinal direction of collagen fibrils,^(^ ^12^ ^)^ we compared cortical structures at discrete positions along the bone axis in the malleus and femur (Fig. 7*A*, *B*, arrows). Specifically, we evaluated the *c*‐axis orientation of biological apatite, an analysis in which higher values indicate more preferential alignment to the bone axis. The maximum degree of *c*‐axis orientation in the mallei (position 3.5) was significantly higher than that found in femur (position 6) (Fig. 7*C*), suggesting that, relative to femoral bone, auditory ossicles are more compact and made of well‐organized collagen fibrils accompanied by apatite crystallites (Fig. 7*D*). Therefore, bone formed by auditory osteoblasts exhibits not only greater mineral content but a higher degree of apatite orientation than that found in conventional osteoblasts.

## Discussion

In this study, we identified a new type of osteoblast (termed auditory osteoblast) and classified osteoblast subtypes based on properties of organic and inorganic bone matrix. The auditory osteoblast forms auditory ossicles, and the bony labyrinth, which are the densest bones in the whole skeleton, exhibit well‐organized apatite crystallite orientation and contain type II collagen as the component of bone matrix. The auditory osteoblast is an osteoblast subtype specialized to form stiff, hearing‐related bones.

### Comparison of auditory with conventional osteoblasts

Our analysis of ossifying auditory ossicles and the bony labyrinth supports the idea that auditory osteoblasts hypermineralize hearing‐related bones. These cells express type II collagen, which conventional osteoblasts do not. Unexpectedly, auditory osteoblasts are GFP‐negative in GFP^OB^ transgenic mice in which conventional osteoblasts express GFP driven by the 2.3 kb‐*Col1* promoter.^(^ ^9^ ^)^ Here, we distinguished auditory from conventional osteoblasts by their lack of GFP signals but expression of osteoblast markers. We note that we detected both endogenous *Col1a1* mRNA and type I collagen protein in GFP‐negative auditory osteoblasts, suggesting that the 2.3 kb‐*Col1a1* promoter carries regulatory elements necessary for transcription in conventional osteoblasts but lacks elements necessary for expression in auditory osteoblasts.^(^ ^29^, ^30^ ^)^ Consistently, others reported that transgenic mice in which the 2.3 kb‐*Col1a1* promoter drove an activate G protein‐coupled receptor showed bone outgrowth surrounding but not in the cochlea itself and not in auditory ossicles.^(^ ^34^ ^)^

Another unexpected finding is that type II collagen expressed in auditory osteoblasts is the component of bone matrix, while conventional osteoblasts primarily express type I collagen. This finding suggests a potential link between type II collagen expression and hypermineralization. Annexin V, a calcium ion transporter found on matrix vesicles, binds with higher affinity to type II than type I collagen,^(^ ^35^ ^)^ suggesting that type II collagen is a more favorable target of matrix vesicles than type I collagen. Consistently, a type II collagen‐coated culture dish more effectively accelerates mineralization by bone marrow–derived osteoblasts compared with a type I collagen‐coated surface.^(^ ^36^ ^)^ Moreover, type II collagen is bound by proteoglycans of higher mass than type I collagen.^(^ ^32^ ^)^ Since calcium ions tend to localize at proteoglycans,^(^ ^37^ ^)^ type II collagen–containing osteoid may enhance mineral incorporation, accounting for the relatively high mineralization of type II collagen‐containing bone.

Lineage tracing experiments have shown that the boundary between chondrocytes and osteoblasts may be more ambiguous than previously thought.^(^ ^38^, ^39^, ^40^ ^)^ We do not exclude the possibility that auditory osteoblasts were originated from the chondrocyte lineage. Regardless of their origin, our results collectively support the idea that auditory osteoblasts are true osteoblasts. They express osteocalcin protein, a marker of mature osteoblasts, at same or higher levels than conventional osteoblasts. They localize along alizarin‐labeled osteoid. They become osteocytes forming lacunae and canaliculi like conventional osteoblasts. In addition, the calcified cartilaginous lattice and bone matrix are morphologically different, indicating that auditory ossicles and bony labyrinth are bone tissues containing type II collagen.

Developmentally, auditory ossicles and the bony labyrinth are derived from the neural crest and mesoderm, respectively,^(^ ^41^ ^)^ suggesting that the origin of auditory osteoblasts is not limited to a single germ layer. A common feature of auditory osteoblasts is their pericytic localization within osteogenic capillaries.^(^ ^9^ ^)^ In this study, we found that chondroclasts are indispensable for generation of auditory osteoblasts. The chondroclast‐dependent differentiation pathway of auditory osteoblasts may be triggered by the osteogenic capillary formation, in which auditory osteoblasts are embedded within the basement membrane.

### Comparison of hearing‐related bones with long bones

Quantification of mineral content in mouse whole skeleton revealed that auditory ossicles and the bony labyrinth are highly mineralized as early as P21. In addition to conventional microCT, we employed qBEI to reveal micrometer scale variation in mineral content in a mineralization front.^(^ ^25^ ^)^ Analysis of the degree of mineralization by qBEI combined with calcein double‐labeling revealed lower bone matrix production in the malleus but a higher mineral content than that found in femur. That analysis also showed high ossicle TMD is due to the high mineral content of extracellular matrix itself, excluding osteocyte lacunae. Relatively decreased bone matrix production is consistent with our finding that auditory osteoblasts are flat‐pericytic cells as opposed to conventional cuboidal osteoblasts that are enriched in rough endoplasmic reticulum. Moreover, stiffness and the relatively high degree of mineralization of auditory ossicles and the bony labyrinth are likely due to their inability to undergo remodeling found in other bones, a property that protects hearing throughout an organism's lifetime.^(^ ^7^, ^42^ ^)^ Our study revealed that hearing‐related bones exhibit high TMD immediately after they are produced. In addition to TMD, bone can exhibit varying parameters such as collagen cross‐linking or apatite orientation.^(^ ^43^ ^)^ We found that the malleus exhibits a higher degree of apatite orientation than do long bones. The higher the TMD and the anisotropic arrangement of apatite crystallites, the more rapid the speed of sound in a bone.^(^ ^44^ ^)^ These data collectively suggest that hypermineralization of auditory ossicles and the bony labyrinth most likely optimizes the material properties required for conductive and sensorineural hearing function.

## Disclosures

All authors state that they have no conflicts of interests.

### PEER REVIEW

The peer review history for this article is available at https://publons.com/publon/10.1002/jbmr.4320.

## Supporting information

**Appendix S1.** Supplemental Information

## Data Availability

Data available on request from the authors.
