# Supplementary figures and images for: Hypermineralization of Hearing‐Related Bones by a Specific Osteoblast Subtype
Source: J Bone Miner Res. 2021 May 14;36(8):1535–47. doi: 10.1002/jbmr.4320 (PMC8453739; doi:10.1002/jbmr.4320)

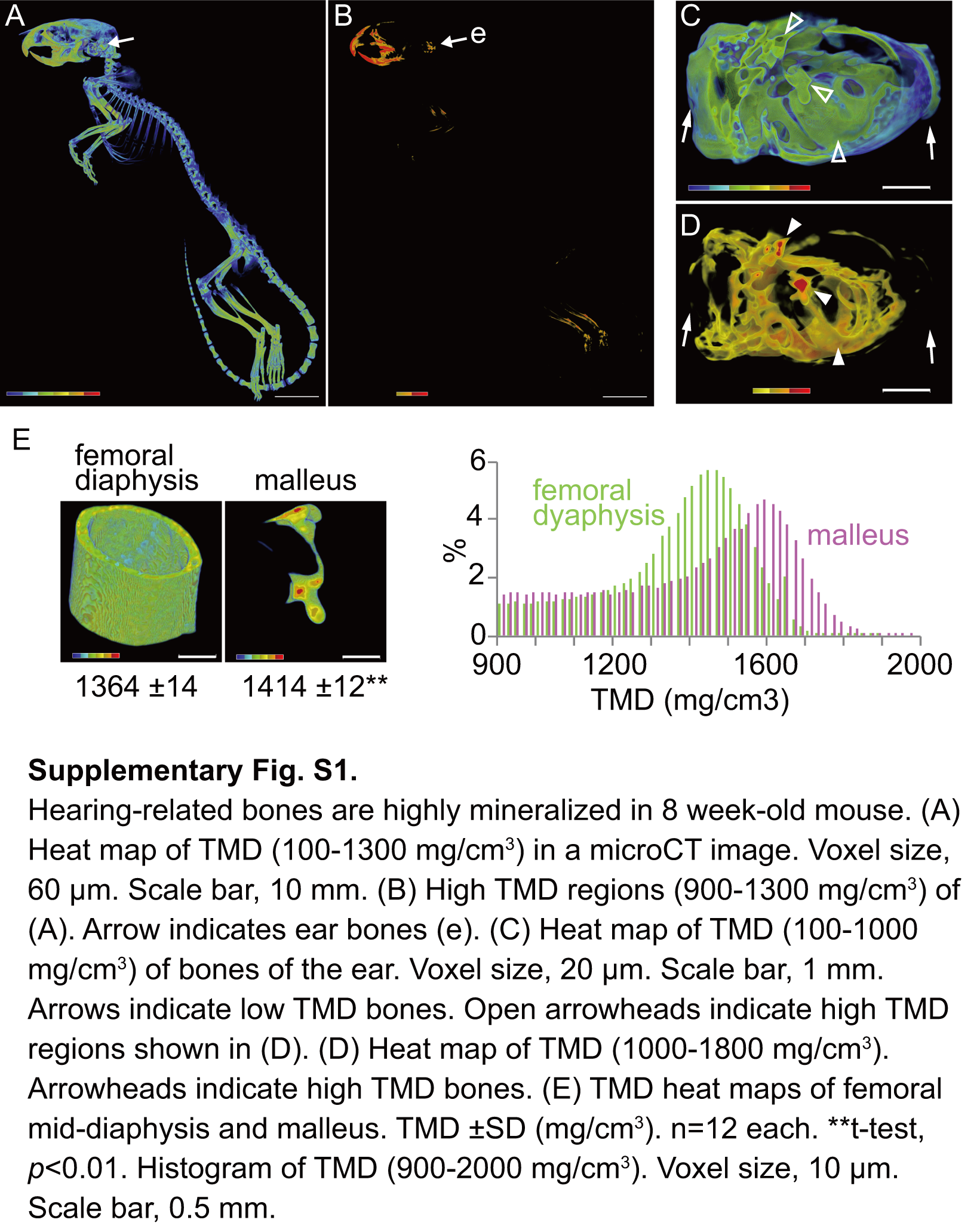

Supplement: Supplementary file 1 — Appendix S1. Supplemental Information [file JBMR-36-1535-s001.zip › jbmr4320-sup-0001-FigureS1.tif]

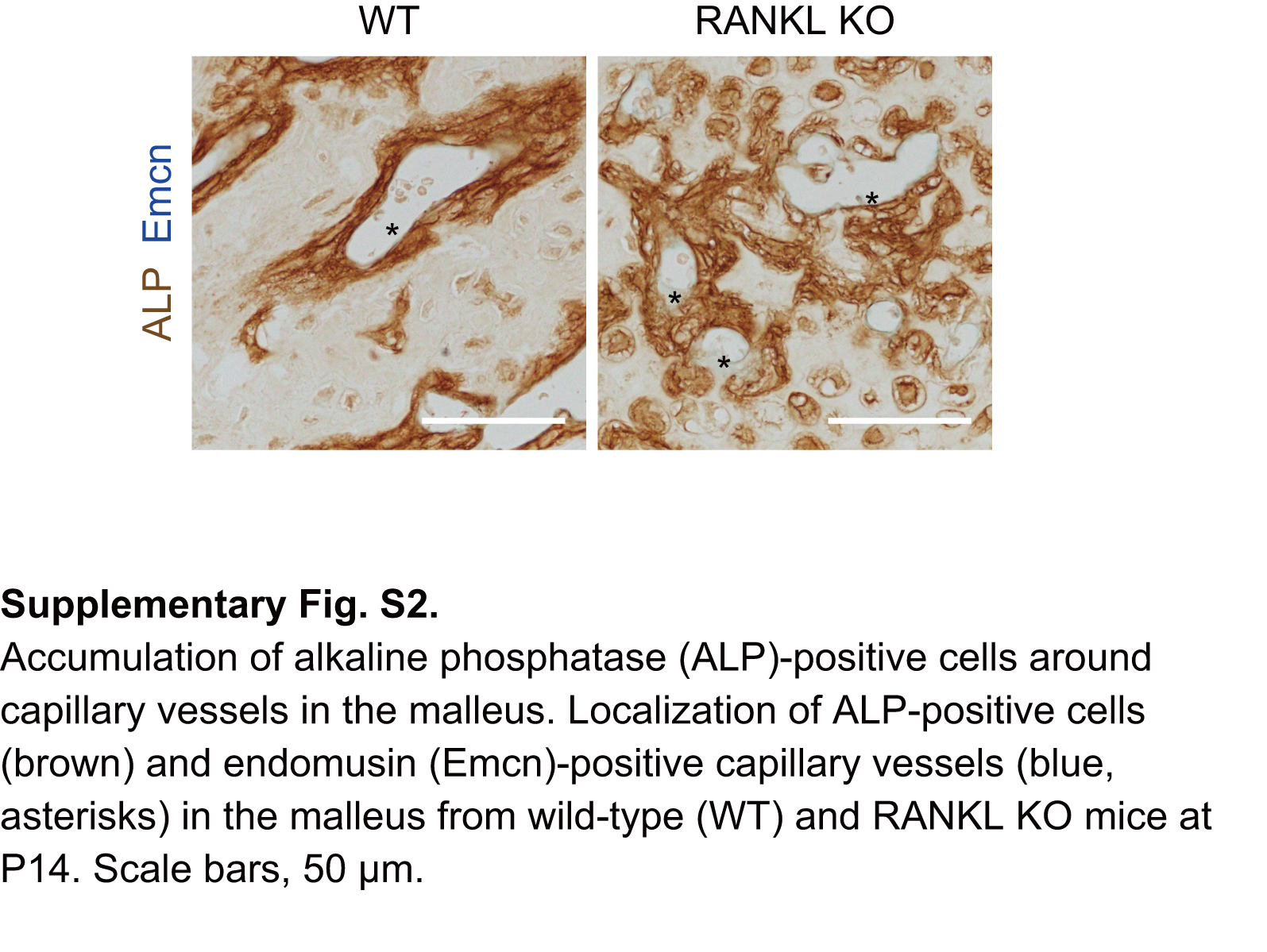

Supplement: Supplementary file 1 — Appendix S1. Supplemental Information [file JBMR-36-1535-s001.zip › jbmr4320-sup-0002-FigureS2.tif]

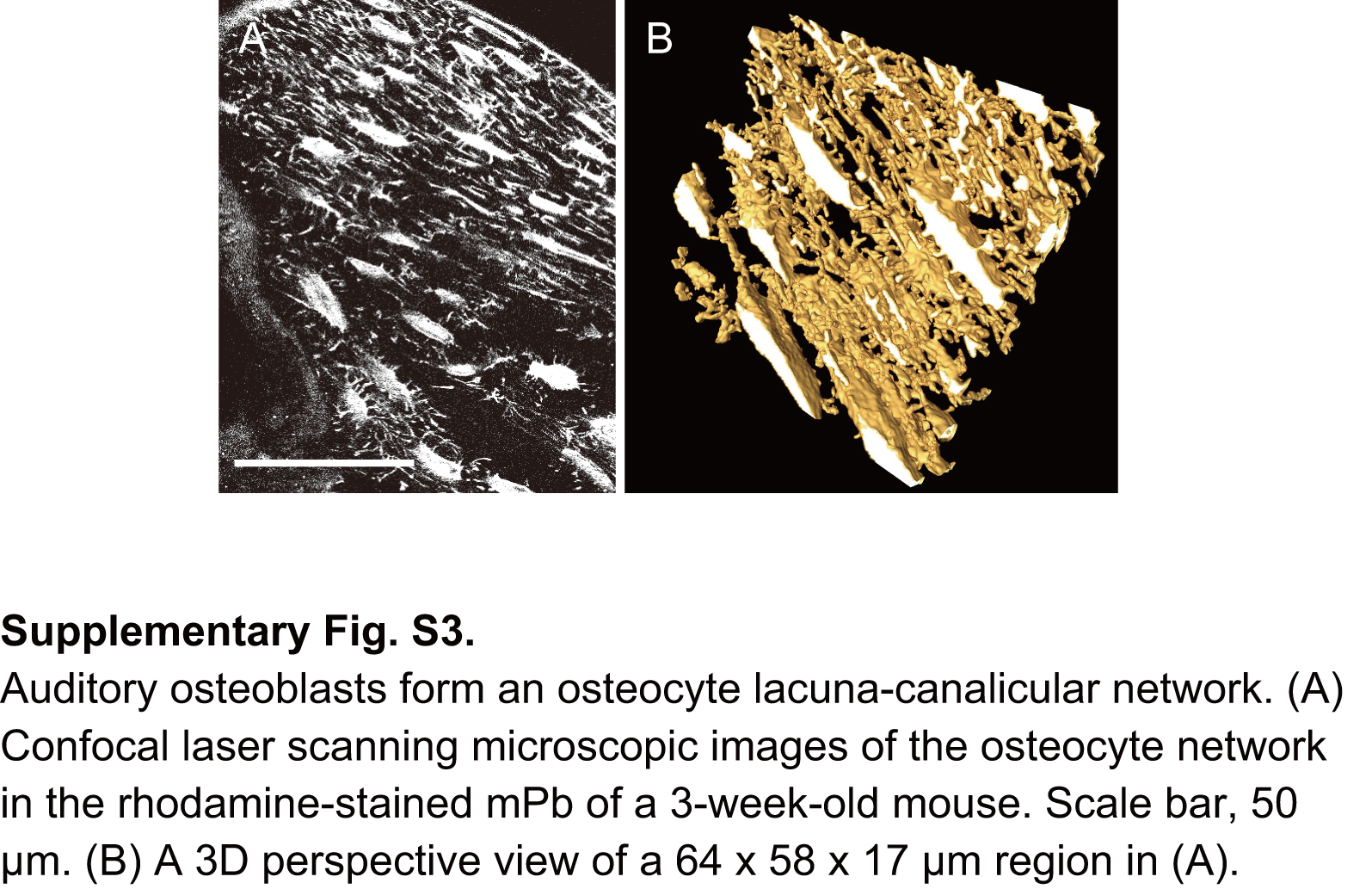

Supplement: Supplementary file 1 — Appendix S1. Supplemental Information [file JBMR-36-1535-s001.zip › jbmr4320-sup-0003-FigureS3.tif]

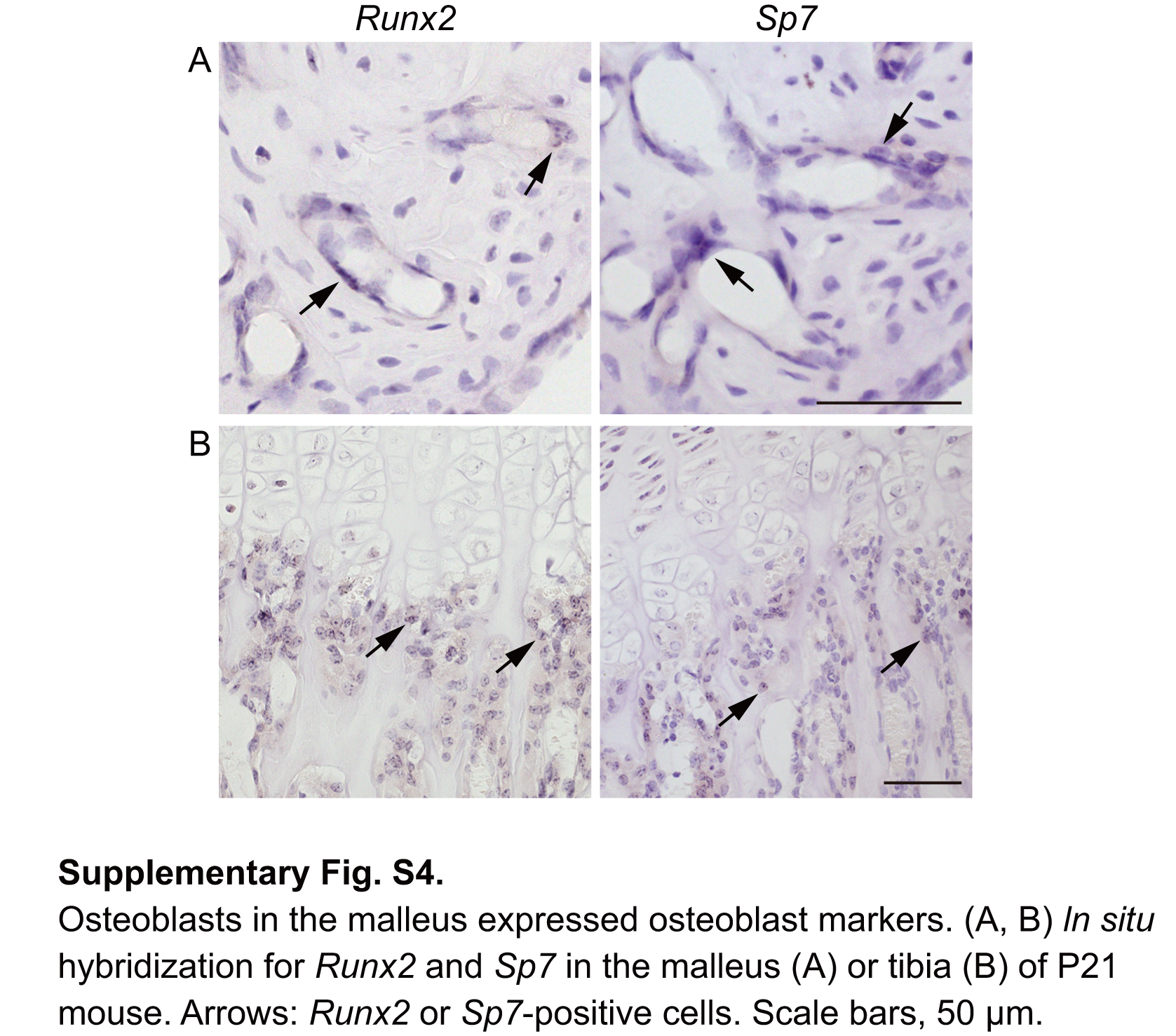

Supplement: Supplementary file 1 — Appendix S1. Supplemental Information [file JBMR-36-1535-s001.zip › jbmr4320-sup-0004-FigureS4.tif]
